# Disseminated peritoneal *Schistosoma japonicum*: a case report and review of the pathological manifestations of the helminth

**DOI:** 10.4103/0256-4947.51800

**Published:** 2009

**Authors:** Salah Al-Waheeb, Maryam Al-Murshed, Fareeda Dashti, Parsotam R. Hira, Lamia Al-Sarraf

**Affiliations:** a Department of Histopathology, Mubarak Al-Kabeer Hospital, Jabriyah, Kuwait; b Department of Surgery, Mubarak Al-Kabeer Hospital, Jabriyah, Kuwait; c Department of Microbiology, Kuwait University, Jabriyah, Kuwait; d Departmment of Radiology, Mubarak Al-Kabeer Hospital, Jabriyah, Kuwait

## Abstract

Schistosomiasis (also known as bilharzia, bilharziasis, bilharziosis or snail fever) is a human disease syndrome caused by infection from one of several species of parasitic trematodes of the genus *Schistosoma.* The three main species infecting humans are *S haematobium, S japonicum, and S mansoni. S japonicum* is most common in the fareast, mostly in China and the Philippines. We present an unusual case of S *japonicum* in a 32-year-old Filipino woman who had schistosomal ova studding the peritoneal cavity and forming a mass in the right iliac fossa.

Schistosomiasis (also known as bilharzia, bilharziasis, bilharziosis or snail fever) is a human disease syndrome caused by infection from one of several species of parasitic trematodes of the genus *Schistosoma.* Approximately 200 million persons are infected with schistosomes worldwide.^1^ Most human schistosomiasis is caused by S *haematobium, S mansoni,* or *S japonicum.* Less prevalent species such as S *mekongi* and S *intercalatum* may also cause systemic human disease. Less importantly, other schistosomes with avian or mammalian primary hosts can cause severe dermatitis in humans (e.g. swimmer's itch secondary to *Trichobilharzia ocellata*). The parasite *S japonicum is* found in the Far East, particularly China and the Philippines.^1^,^2^ The disease manifestations caused *by S. japonicum* in humans have been extensively covered in the literature and will be reviewed in this paper. We present an unusual case of *S japonicum* where the patient had schistosomal ova studding the peritoneal cavity and forming a mass in the right iliac fossa (RIF).

## CASE

**Figure 1:**
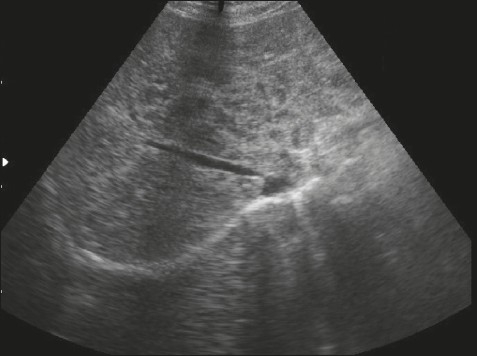
Ultrasound of the liver shows heterogenous echogenicity.

**Figure 2:**
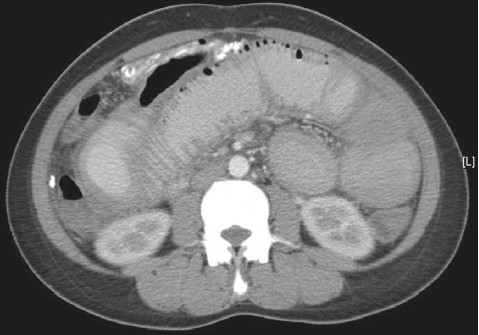
CT scan shows multiple dilated small bowel loops with multiple calcific peritoneal deposits.

**Figure 3:**
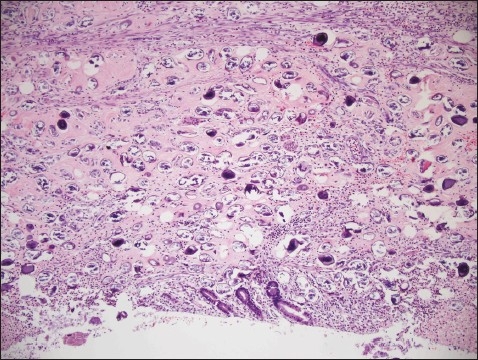
Appendiceal wall studded with schistosomal ova (hematoxylin and eosin stain ×200).

**Figure 4:**
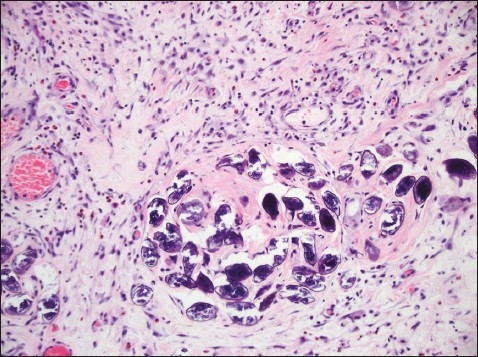
*Schistosoma Japonicum* ova with characteristic lateral outpouchings (hematoxylin and eosin stain ×200).

A 32-year-old Filipino woman presented with a 5-day history of generalized abdominal pain and a 3-day history of nausea and vomiting. The pain settled in the RIF. She was admitted with a WBC count of 11×10^9^/L and a temperature of 38.5° C. Ultrasound studies of the abdomen showed heterogeneous echogenicity of the liver (Figure 1). CT examination showed multiple calcific foci throughout the abdomen, particularly in the RIF. Prominent small bowel dilatation and fluid collection in the pouch of Douglas were also noted (Figure 2). The liver appeared surprisingly normal on the CT study. Diagnostic laparoscopy was immediately converted to an open laparotomy once a RIF mass with pus involving the appendix was seen. Dilated small bowel loops were present in the RIF and next to the spleen. The mass was dissected and released and the appendix was removed, after which the small intestinal obstruction subsided. Intraoperatively, multiple whitish yellow peritoneal nodules were noted covering the abdominal peritoneal surface and the surface of the liver. The liver, however, did not appear to be cirrhotic. On gross examination, the appendix was severely congested, measured 6×2×1 cm and showed an area of perforation at the body. The adherent omentum had focal areas of pus, and in other areas, firm yellow white nodules were present. The microscopic sections showed the appendiceal wall studded with schistosomal ova (Figure 3). Furthermore polymorphonuclear leukocytes were also seen in the wall along with areas of suppurative inflammation and necrosis. Sections from the adherent omental mass also showed suppurative inflammation and multiple foci of shistosomal ova. The ova were oval to round in shape and showed outpouchings highly indicative of *S japonicum* species (Figure 4). No granulomatous response was present in the sections examined. Stains for bacterial, fungal and acid fast organisms were all negative. The histopathologic diagnosis was acute suppurative appendicitis and peritonitis with disseminated schistosomal ova involving the appendix and omentum.

## DISCUSSION

From a clinical perspective *S japonicum* usually causes an acute serum sickness also called ‘Katayama’ fever,^3^ which is associated with the onset of the female parasite laying eggs (approximately 5 weeks after infection) and granuloma formation around eggs trapped in the liver and intestinal wall. It manifests with hepatosplenomegaly and leucocytosis with eosinophilia. This phase of the infection is often asymptomatic, but when symptoms do occur they include fever, nausea, headache, an irritating cough and, in extreme cases, diarrhea accompanied with blood, mucus and necrotic material. These symptoms, if present, last from a few weeks, to several months and are not as commonly associated with S *hematobium* or S *mansoni* infections compared with those of *S japonicum.* The chronic phase of infection, however, is the more important part of the infection.^3^ From a pathological perspective, common morphological manifestations of the chronic phase are intestinal and hepatic schistosomiasis. Both manifest a number of years after infection. A large autopsy study involving 349 cases showed that the dominant pathology is evident in the liver.^4^ The pathogenic reaction is a cellular, granulomatous inflammation around eggs trapped in the tissues, with subsequent fibrosis. Specific liver pathology was destruction of limiting plates, reparative hepatic lesions such as regeneration of the collapsed parenchyma, newly formed limiting plates and subsequent narrowing and disappearance of fibrous septa. In more chronic lesions, fibrosis and cirrhosis are seen. Complications of liver cirrhosis and hepatocellular carcinoma related to viral hepatitis B and/or C were also increased. Clonorchiasis was also consistently found.^4^ All areas of both the small and large intestine may be involved, with the large intestine showing the most severe lesions, whereas severe pathology in the small intestine is only rarely observed, even though a large number of eggs may be deposited there.^5^ The theory is that the adult worms have a predilection for inhabiting the branches of the inferior mesenteric vein and superior haemorrhoidal vein and their eggs are deposited in much higher density in the large intestine, especially in the rectum, sigmoid and descending colon, than in the small intestine.^5^ Gastrointestinal findings may include polyp formation. The friable vascular nature of these inflammatory polyps may lead to lower gastrointestinal bleeding.^6^ Large polyps, or bilharziomas, may cause intestinal obstruction, intussusception, or be confused with a malignancy.^7^ Colonic polyps are of higher prevalence in Egypt for reasons that are not clear.^8^ Other cited gastrointestinal findings may include strictures, fistulae and bowel perforation.^7^,^9^ *S japonicum* eggs may also be deposited in the appendix and may manifest as appendicitis. Schistosomal appendicitis acquired in a traveler has been described.^10^

Liver-related and gastrointestinal complications can be prevented with early recognition and therapy of schistosomiasis. In a radiologic study of 12 patients that underwent CT of the abdomen to detect what proved to be *S japonicum* by pathologic examination, the CT demonstrated curvilinear or nodular calcification in the colon in 11 patients, in the appendix in 2, and in the distal ileum in 1 patient. Pathologic examination of the specimens showed calcified eggs of *S japonicum* deposited more extensively in the submucosa and subserosa than in the muscularis propria, which led to the curvilinear appearance.^6^ The pathology in the liver is similar to that seen in the intestine, with a cellular, granulomatous inflammation around eggs trapped in the liver, leading to fibrosis and hepatosplenic disease and subsequent cirrhosis in chronic disease. Studies on the effect of *S japonicum* and the liver have been recorded since the early 1970s from endemic areas.

Kurniawan et al studied 52 *S japonicum*-infected patients from an endemic area in Indonesia.^11^ All of these patients exhibited signs and symptoms of chronic hepato-splenic schistosomiasis. None of the patients showed evidence of liver cirrhosis on histopathological examination. However, varying degrees of portal fibrosis were exhibited and the authors concluded that liver biopsy has proved to be a valuable method of diagnosis in this particular type of infection. *S japonicum* resides in the mesenteric veins which drain to the liver, and therefore causes liver fibrosis after depositing in that organ.^12^ A study of the ultrasound and CT scan findings of patients with biopsy-proven hepatic *S japonicum* revealed characteristic findings.^13^ They observed a “network pattern of calcification” on ultrasound and a “turtle-back” pattern on contrast CT scan. Laparoscopic findings of nine patients with chronic *S japonicum* were analyzed and compared with histological findings from the same patients.^14^ In all nine patients laparoscopy revealed yellowish, small speckles clustered over the liver surface, which were later found to be the subcapsular calcified ova of *S japonicum.* While the liver surface was almost smooth in mild schistosomiasis, multiple whitish markings and irregular, relatively wide, groove-like septums were seen in more advanced cases. In severe schistosomiasis, block-like formations of variable size, separated by groove-like depressions, made the liver surface appear like a tortoise shell on the CT scan. Other organs may rarely contain granulomas around eggs, like the Breast^15^ and fallopian tube.^16^

What is unusual and novel about our case from a morphologic perspective is that there has not been a reported case in the English language literature of *S japonicum* disseminating and covering the peritoneum in this. The patient was lost to followaup and no colonoscopy was done to rule out any colonic pathology which may have been present in the patient. Similarly, the liver might have been involved with *S japonicum* ova, as suspicious nodular lesions were seen studding the surface of the liver intraoperatively, but no liver biopsy was performed. The liver enzymes were normal in the patient and no overt liver cirrhosis was present. The other unusual feature was the lack of a granulomatous reaction in the tissue examined in our laboratory.
